# Antifibrotic Effects of a Barbituric Acid Derivative on Liver Fibrosis by Blocking the NF-κB Signaling Pathway in Hepatic Stellate Cells

**DOI:** 10.3389/fphar.2020.00388

**Published:** 2020-03-31

**Authors:** Yuan-Hsi Wang, Fat-Moon Suk, Chao-Lien Liu, Tzu-Lang Chen, Yuh-Ching Twu, Ming-Hua Hsu, Yi-Jen Liao

**Affiliations:** ^1^Department of Biotechnology and Laboratory Science in Medicine, School of Biomedical Science and Engineering, National Yang-Ming University, Taipei, Taiwan; ^2^School of Medical Laboratory Science and Biotechnology, College of Medical Science and Technology, Taipei Medical University, Taipei, Taiwan; ^3^Division of Gastroenterology, Department of Internal Medicine, Wan Fang Hospital, Taipei Medical University, Taipei, Taiwan; ^4^Department of Internal Medicine, School of Medicine, College of Medicine, Taipei Medical University, Taipei, Taiwan; ^5^Department of Medical Education, Far Eastern Memorial Hospital, New Taipei City, Taiwan; ^6^Department of Chemistry, National Changhua University of Education, Changhua, Taiwan

**Keywords:** barbituric acid derivative, fibrosis, hepatic stellate cells, liver, NF-κB signaling, TGF-β1/Smad, *in vitro* study, animal model

## Abstract

Hepatic stellate cells (HSCs) are the major profibrogenic cells that promote the pathogenesis of liver fibrosis. The crosstalk between transforming growth factor-β1 (TGF-β1) signaling and lipopolysaccharide (LPS)-induced NF-κB signaling plays a critical role in accelerating liver fibrogenesis. Until now, there have been no FDA-approved drug treatments for liver fibrosis. Barbituric acid derivatives have been used as antiasthmatic drugs in the clinic; however, the effect of barbituric acid derivatives in treating liver fibrosis remains unknown. In this study, we synthesized a series of six barbituric acid (BA) derivatives, and one of the compounds, BA-5, exhibited the best ability to ameliorate TGF-β1-induced HSC activation without overt cytotoxic effects. Then, we treated HSCs and RAW264.7 macrophages with BA-5 to analyze the cross-talk of anti-fibrotic and anti-inflammatory effects. Carbon tetrachloride (CCl_4_)-induced liver fibrosis mouse model was used to evaluate the therapeutic effects of BA-5. Treatment with BA-5 inhibited TGF-β1-induced α-SMA, collagen1a2, and phosphorylated smad2/3 expression in HSCs. Furthermore, BA-5 treatment reversed the LPS-induced reduction in BAMBI protein and decreased IκBα and NF-κB phosphorylation in HSCs. NF-κB nuclear translocation, MCP-1 secretion, and ICAM-1 expression were also inhibited in BA-5-treated HSCs. Conditioned medium collected from BA-5-treated HSCs showed a reduced ability to activate RAW264.7 macrophages by inhibiting the MAPK pathway. In the mouse model, BA-5 administration reduced CCl_4_-induced liver damage, liver fibrosis, and F4/80 expression without any adverse effects. In conclusion, our study showed that the barbituric acid derivative BA-5 inhibits HSCs activation and liver fibrosis by blocking both the TGF-β1 and LPS-induced NF-κB signaling pathways and further inhibits macrophages recruitment and activation.

## Introduction

Liver fibrosis is a pathological response of the liver to a variety of chronic diseases, such as alcohol consumption, non-alcoholic steatohepatitis, non-alcoholic fatty liver disease, viral hepatitis, autoimmune hepatitis, and cholestatic liver diseases (Campana and Iredale, 2017; Aydin and Akcali, 2018). Liver fibrosis is characterized by the surplus accumulation of extracellular matrix, which follows chronic liver injury (Trautwein et al., 2015). If the injury persists, liver fibrosis can progress to cirrhosis and ultimately result in the development of liver cancer (Affo et al., 2017). Hepatic stellate cells (HSCs) are the major cell type responsible for liver fibrogenesis (Barcena-Varela et al., 2019). Upon liver injury, HSCs become activated and transdifferentiate into myofibroblast‐like cells that proliferate and migrate to regions of hepatocyte injury and produce collagen and α‐smooth muscle actin (α‐SMA) (Barcena-Varela et al., 2019). HSC activation is driven by multiple mediators, such as transforming growth factor-β1 (TGF-β1), platelet-derived growth factor, and tumor necrosis factor-α (Dewidar et al., 2019). Among these, TGF-β1 is the most potent mediator in accelerating liver fibrosis by activating smad2/3 signaling (Hu et al., 2018). Therefore, TGF-β1 signaling represents a potential therapeutic target for treating liver fibrosis.

Previous studies indicate that the lipopolysaccharide (LPS)/Toll-like receptor 4 (TLR4) signaling pathway plays an important role in liver fibrogenesis (Liu et al., 2014; Kiziltas, 2016). In hepatic fibrosis mouse models and cirrhosis patients, bacterial translocation and LPS levels in the liver were increased (Pradere et al., 2010). LPS activates TLR4 signaling through the adaptor protein MyD88 to activate the downstream NF-κB signaling pathway and further downregulate expression of the TGF-β1 pseudoreceptor BAMBI (Kiziltas, 2016). Therefore, LPS sensitizes HSCs to enhance TGF-β1-induced signaling and further enhances HSC activation (Petrasek et al., 2013; Kiziltas, 2016). In addition, TLR4 activation also leads to MCP-1 secretion and ICAM-1 upregulation in HSCs, thereby guiding monocyte recruitment to the liver (Tacke and Zimmermann, 2014). Many studies have demonstrated that hepatic macrophage infiltration promotes liver fibrosis through the recruitment of immune cells and the secretion of cytokines and chemokines (Wynn and Barron, 2010; Tacke and Zimmermann, 2014; Li et al., 2016). Although several new compounds have been developed in recent years (Levada et al., 2019), no antifibrotic therapy has yet been approved by the FDA. Therefore, modulation of TLR4-MyD88-NF-κB signaling might represent a feasible strategy for the treatment of liver fibrosis.

Barbituric acid is an organic compound based on a pyrimidine heterocyclic skeleton and was first discovered by the German chemist Adolf von Baeyer in 1864 (Baeyer, 1864). Although barbituric acid is not pharmacologically active, it can form a large variety of derivatives called barbiturates that have been used in many ways (Shahzad et al., 2016). Barbituric acid derivatives possess different biological activities, such as hypnotic (Shonle and Moment, 1923; Wisner, 1925), sedative (Kliethermes et al., 2004), anticonvulsant (Srivastava and Kumar, 2004), antimicrobial (Dhorajiya et al., 2014), antiviral (Marecki et al., 2019), anti-inflammatory (Xu et al., 2016), anticancer, and antitumor properties (Singh et al., 2009; Penthala et al., 2015; Laxmi et al., 2016). In 2011, a study showed that a novel barbituric and thiobarbituric acid derivative inhibited high-fat/high-calorie diet-induced non-alcoholic fatty liver disease in male Wistar rats (Ma et al., 2011). These results provide the impetus for us to develop novel and potent therapeutic agents containing barbituric acid and to investigate their pharmacological functions in treating liver fibrosis. Therefore, the purpose of this study was to identify the effect of novel barbituric acid derivative on the TGF-β1 and LPS-induced NF-κB signaling pathways in activated hepatic stellate cells and further influences macrophages recruitment by using both *in vitro* and *in vivo* experiments.

## Materials and Methods

### Barbituric Acid Derivative Synthesis

#### Chemistry

All reactions were carried out in oven-dried glassware (120°C) under an atmosphere of nitrogen, unless as indicated otherwise. Acetone, dichloromethane, ethyl acetate, hexane, and tetrahydrofuran were purchased Mallinckrodt Chemical Co. Acetone was dried with 4Å molecular sieves and distilled. Dichloromethane, ethyl acetate, and hexane were dried and distilled from CaH2. Tetrahydrofuran were dried by distillation from sodium and benzophenone under an atmosphere of nitrogen. Melting point was obtained with a Buchi 535 melting point apparatus. Analytical thin layer chromatography (TLC) was performed on precoated plates (silica gel 60 F–254), purchased from Merck Inc. Ultraviolet (UV) spectra were measured on Hitachi U3300 UV/VIS spectrophotometer. Infrared (IR) spectra were measured on Jasco FT–IR-5300 Fourier transform infrared spectrometer. The wave numbers reported are referenced to the polystyrene 1601 cm–1 absorption. Absorption intensities are recorded by the following abbreviations: s, strong; m, medium; w, weak. 1H NMR spectra were obtained on a Varian Mercury-400 (400 MHz) spectrometer by use of DMSO-d6 as the solvent and sodium 3-(trimethylsilyl) propionate as internal standard. 13C NMR spectra were obtained on a Varian Mercury-400 (100 MHz) spectrometer by use of DMSO-d6 as the solvent. Multiplicities are recorded by the following abbreviations: s, singlet; d, doublet; t, triplet; q, quartet; m, multiplet; J, coupling constant (hertz).

#### Standard Procedure for the Syntheses of Barbiturate-Conjugated Derivatives

**Scheme 1:**
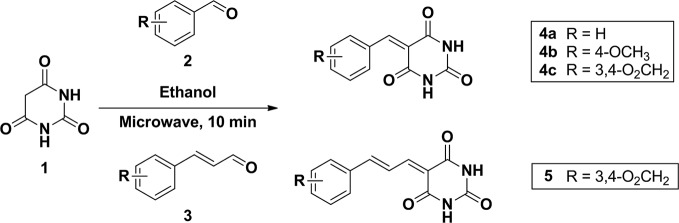
Synthesis of compound 4a, 4b, 4c, and 5.

**Scheme 2:**
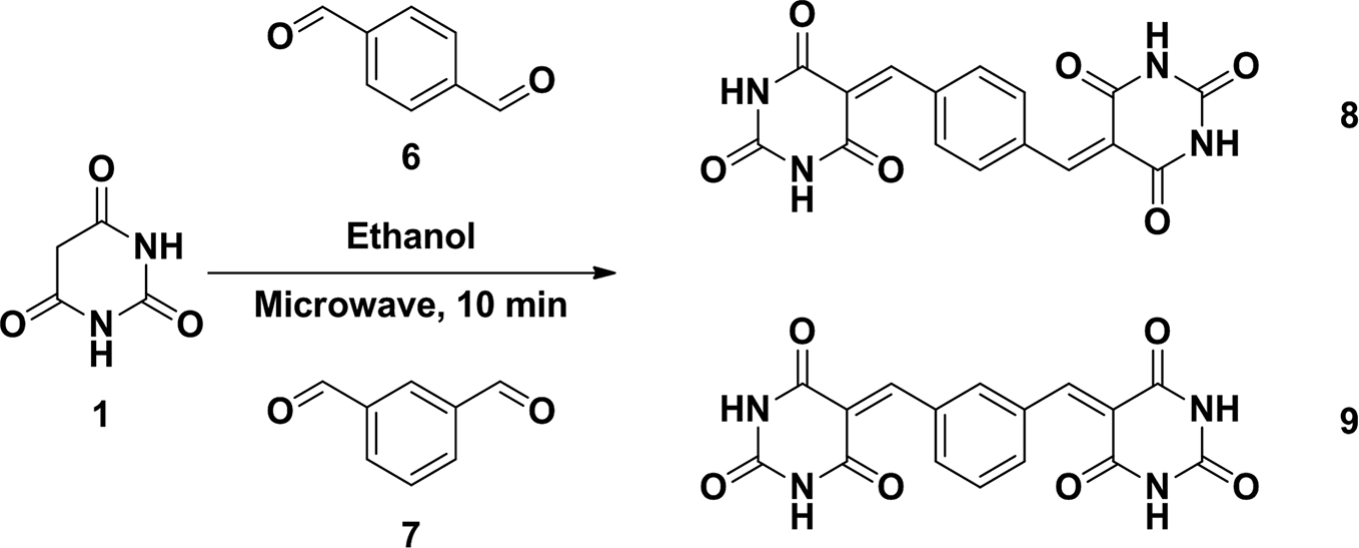
Synthesis of compound 8 and 9.

To a mixture of barbituric acid (0.5g, 3.9 mmol) and arylaldehyde (3.9 mmol) in ethanol (5.0 ml) were set into a microwave reaction vial tube. The mixture was heated in microwave reactor at 140°C for 10 min. After the reaction cool down to room temperature, the mixture was poured into 15 ml water causing product precipitated and stir for 10 min. The precipitate was collected and washed by water twice to give corresponding compounds. All the compounds were confirmed by 1H NMR and the purity was checked by HPLC (**Schemes 1** and **2**).

##### 5-benzylidenepyrimidine-2,4,6(1H,3H,5H)-trione (4a)

Its spectroscopic characteristics are consistent with those of the same compound reported (Khalafi-Nezhad and Hashemi, 2001).

##### 5-(4-methoxybenzylidene)pyrimidine-2,4,6(1H,3H,5H)-trione (4b)

1H NMR (DMSO- d6; 400MHz), δ: 3.88 (s, 3H, OCH3), 7.06 (d, J = 8.8Hz, 2H, Ar-H), 8.25 (s, 1H, CH=), 8.37 (d, J = 8.8Hz, 2H, Ar-H), 11.18 (s, 1H, N-H), 11.30 (s, 1H, N-H). MS (ES), m/z: 231.2. Its spectroscopic characteristics are consistent with those of the same compound reported (Luo et al., 2010).

##### 5-(benzo[d][1,3]dioxol-5-ylmethylene)pyrimidine-2,4,6(1H,3H,5H)-trione (4c)

1H NMR (DMSO-d6; 400MHz), δ: 6.170 (s, 2H, O2CH2), 7.069 (d, J = 8.4 Hz, 1H, Ar-H), 7.729 (d, J = 8.4 Hz, 1H, Ar-H), 8.194 (s, 1H, Ar-H), 8.246 (s, 1H, CH=), 11.207 (s, 1H, N-H), 11.323 (s, 1H, N-H). MS (ESI): m/z, 277.050 (ES+).

##### (E)-5-(2-methyl-3-phenylallylidene)pyrimidine-2,4,6(1H,3H,5H)-trione

(5): 1H NMR (DMSO-d6; 400MHz), δ: 2.086 (s, 3H, CH3), 3.949 (s, 1H, CH=C), 4.192 (s, 1H, CH=), 6.492 (s, 1H, Ar-H), 7.017~7.101 (m, 2H, Ar-H), 7.180 (m, 2H, Ar-H), 7.216 (s, 1H, Ar-H), 11.009 (s, 1H, N-H), 11.586 (s, 1H, N-H).

##### 5,5′-(1,4-phenylenebis(methanylylidene))bis(pyrimidine-2,4,6(1H,3H,5H)-trione) (8)

1H NMR (DMSO- d6; 400MHz), δ: 8.034 (s, 4H, Ar-H), 8.273 (s, 2H, CH=), 11.288 (s, 2H, N-H), 11.436 (s, 2H, N-H). MS (ESI): m/z, 355.067 (ES+).

##### 5,5′-(1,3-phenylenebis(methanylylidene))bis(pyrimidine-2,4,6(1H,3H,5H)-trione) (9)

1H NMR (DMSO- d6; 400MHz), δ: 7.541 (t, J = 7.2Hz, 1H, Ar-H), 8.187 (d, J = 8.8Hz, 2H, Ar-H), 8.264 (s, 1H, Ar-H), 8.472 (s, 2H, CH=), 11.275 (s, 2H, N-H), 11.430 (s, 2H, N-H). MS (ESI): m/z, 355.068 (ES+).

### Cell Culture

LX2 cells and HSC-T6 cells (an immortalized strain of rat HSCs) (Twu et al., 2016) were cultured in Dulbecco’s modified Eagle’s medium (DMEM; Gibco BRL, Grand Island, NY, USA) with 1% heat-inactivated fetal bovine serum (FBS; HyClone, Logan, UT, USA), penicillin and streptomycin (100 U/ml), nonessential amino acids (0.1 mM), and L-glutamine (2 mM) at 37°C in a 5% CO_2_ incubator. Murine RAW264.7 macrophages (Bioresource Collection and Research Center, BCRC 60001, Taiwan) were cultured in DMEM supplemented with 10% FBS, penicillin and streptomycin (100 U/ml), nonessential amino acids (0.1 mM), and L-glutamine (2 mM) at 37°C in a 5% CO_2_ incubator.

### AlamarBlue Cell Viability Assay

Cell viability was determined by the commercial alamarBlue cell viability reagent (Life Technologies, Carlsbad, CA, USA) which functions as a cell health indicator by using the reducing power of living cells to quantitatively measure the proliferation of cultured cells (O’Brien et al., 2000). LX2 cells were seeded (2 × 10^4^) in a 96-well plate. After 24 and 48 h, 10 μl of alamarBlue reagent was added to 100 μl culture medium and incubated for 2.5 h at 37°C in a 5% CO_2_ incubator. The nontoxic alamarBlue (resazurin) enters living cells and is reduced by mitochondrial FMNH2, FADH2, NADH, NADPH, and cytochromes. The reagent transforms from blue nonfluorescent resazurin to pink fluorescent resorufin. The absorbance was measured at 570 nm, and we used 600 nm as the reference wavelength (normalized to the 600 nm value) to quantitatively measure the metabolically active cells.

### TGF-β1 Treatment

To study the effect of BA-5 on TGF-β1-induced fibrogenesis, LX2 cells were (1) seeded in 6-well plates (1 × 10^5^ per well) and treated with 0, 1.25, 2.5, and 5 μM BA-5 and 10 ng/ml TGF-β1 (R&D Systems, Minneapolis, MN, USA) for 24 h; and (2) seeded in 6-well plates (1 × 10^5^ per well), pretreated with or without 5 μM BA-5 for 24 h and further treated with 10 ng/ml TGF-β1 for 0, 15, 30, and 60 min. HSC-T6 cells were (1) seeded in 6-well plates (2.5 × 10^5^ per well) and treated with 5 μM BA-5 and 10 ng/ml TGF-β1 for 24 h; and (2) seeded in 6-well plates (2 × 10^5^ per well), pretreated with or without 5 μM BA-5 for 24 h and further treated with 10 ng/ml TGF-β1 for 30 min.

### LPS Treatment

To study the effect of BA-5 on LPS-induced inflammation, LX2 cells were seeded in 6-well plates (1 × 10^5^ per well), pretreated with or without 5 μM BA-5 for 24 h and further treated with 100 ng/ml LPS (Sigma-Aldrich, St Louis, MO, USA) for 30 min.

### Immunofluorescence Staining

LX2 cells were seeded in 24-well plates (2 × 10^4^ per well), pretreated with or without 5 μM BA-5 for 3 h and further treated with 100 ng/ml LPS for 1 h. The culture medium was removed and then the cells were fixed in 4% paraformaldehyde for 30 min. After permeabilization with 0.5% Triton X-100 for 5 min at room temperature, the cells were blocked with 0.01 g/ml albumin for 1 h. Subsequently, the cells were incubated with antibodies against total-p65 (1:100; Cell Signaling, Beverly, MA, USA) overnight at 4 °C. Then, the LX2 cells were incubated with anti-IgG (1:200) for 1 h at room temperature. Finally, the cells were counterstained with 4′,6-diamidino-2-phenylindole (DAPI) (1:600; Molecular Probes, Eugene, OR, USA) before capturing images by using a fluorescence microscope (Nikon Eclipse TE 2000-U, Tokyo, Japan).

### Nuclear/Cytosolic Fractionation

LX2 cells were seeded in 6-well plates (1 × 10^5^ per well), pretreated with or without 5 μM BA-5 for 3 h and further treated with 100 ng/ml LPS for 1 h. Nuclear/cytosolic fractionation was measured by using a commercial colorimetric kit (BioVision, Mountain View, CA, USA). Nuclear/cytosolic fractionation was carried out according to the manufacturer’s instructions.

### ELISA

LX2 cells were seeded in 6-well plates (1 × 10^5^ per well), pretreated with or without 5 μM BA-5 for 24 h and further treated with 100 ng/ml LPS for 9 h. R&D ELISA kits (MCP-1, IL-6, and TNF-α) were used to measure the cytokine content of cell supernatants. ELISAs were carried out according to the manufacturer’s instructions (R&D Systems, Minneapolis, MN, USA).

### Production of HSC‐Derived Conditioned Medium

LX2 cells were pretreated with 5 μM BA-5 overnight and further treated with 100 ng/ml LPS for 9 h. The conditioned media were collected and centrifuged at 1500 rpm for 5 min, and the supernatant fractions were transferred to new tubes. The generated conditioned media were kept at -80°C until use.

### Animal Model

C57BL/6 male mice, aged 7–8 weeks, were purchased from the Taiwan National Laboratory Animal Center. All mice were maintained on a standard chow diet (No. 5001, LabDiet, St Louis, MO, USA) and housed in conditions with a 12-h light-dark cycle. The fibrosis model was generated using carbon tetrachloride (CCl_4_, Sigma-Aldrich, St Louis, MO, USA). The mice were assigned randomly to four groups: (1) vehicle control (0.9% Normal Saline; daily intraperitoneal injection for 7 weeks), n=5, (2) CCl_4_ (2 ml/kg; twice-weekly intraperitoneal injection for 6 weeks), n=6, (3) BA-5 (4 mg/kg; daily intraperitoneal injection for 7 weeks), n=5, and (4) CCl_4_ plus BA-5 (administered as previously described; BA-5 pretreatment for 1 week and co-treatment with CCl_4_ for 6 weeks), n=6. All animal experiments were carried out in accordance with the guidelines of the National Institute of Heath’s Guide for the Care and Use of Laboratory Animals, and were approved by the Institutional Animal Care and Use Committee of Taipei Medical University (LAC-2017-0476). Mouse body weight was monitor once weekly by using an electronic balance. Liver tissues and serum were collected at the end of the experiments. The samples used in protein and RNA analyses were frozen in liquid nitrogen and stored at -80°C, while those used in IHC staining were fixed in 10% formalin.

### Immunohistochemistry (IHC) Staining and Blood Biochemical Parameters

The liver from each mouse was removed and fixed in freshly prepared 10% formalin. The sections were stained with hematoxylin and eosin (H&E) for histopathological examination. Sirius red staining (Abcam, Cambridge, MA, USA) of paraffin-embedded liver sections was used to qualitatively assess the collagen architecture and the extent of fibrosis in accordance with the manufacturer’s instructions. Masson’s Trichrome staining (Abcam, Cambridge, MA, USA) was carried out in accordance with the manufacturer’s instructions to investigate the collagen architecture. Paraffin-embedded liver sections were incubated with the antibodies against F4/80 (1:200; Cell Signaling, Beverly, MA, USA) and detected using the Universal LSABTM2 kit (DakoCytomation, Carpinteria, CA, USA) according to the manufacturer’s instructions. All sections were investigated by a light microscope (Olympus CKX41, Olympus Corp., Tokyo, Japan). Histopathological diagnosis of the mouse liver tissue was analyzed by gastroenterological physician. Serum alanine aminotransferase (ALT), albumin (ALB), and blood urea nitrogen (BUN) values were measured with a biochemical analyzer (VetTest™, IDEXX, USA).

### Western Blotting

Isolation of total protein from cultured cells was performed by using lysis buffer supplemented with protease and phosphatase inhibitors. The protein concentration was measured by protein assay (BioRad, Hercules, CA, USA), and all samples were normalized to 30 μg. Cellular proteins were separated by SDS-PAGE and transferred onto polyvinylidene difluoride (PVDF) membranes. Primary and secondary antibodies were used to label the target on the membrane. The α-SMA (ab5694) and BAMBI (ab57043) antibody used in this study were purchased from Abcam (Cambridge, MA, USA). The following antibodies were purchased from Cell Signaling (Beverly, MA, USA): T/p (Ser465/467; Ser423/425)-smad2/3 (#8685, #8828), T/p (Ser536)-p65 (#8242, #3033), T/p (Ser176/180)-IKKα/β (#11930, #8943, #2697), T/p (Ser32)-IκBα (#4814, #2859), T/p (Thr202/Tyr204)-ERK (#4695, #9101), T/p (Thr183/Tyr185467)-JNK (#9252, #4668), and T/p (Thr180/Tyr182)-p38 (#8690, #4511). The immunoblotting signals were normalized to α-tubulin (T9026) (Sigma-Aldrich, St Louis, MO, USA). The bands were visualized using an ECL detection reagent (Millipore Corporation, Billerica, MA, USA).

### RNA Extraction and Real-Time PCR

Total RNA was isolated from cultured cells by using TRIzol reagent (Ambion, Carlsbad, CA, USA) according to the manufacturer’s protocol. High-capacity cDNA reverse transcription kits (Applied Biosystems, Carlsbad, CA, USA) were used to produce complementary DNA from cellular RNA (2 μg). A total of 4 µl template cDNA (20 ng), 5 µl KAPA SYBR^®^ FAST qPCR master mix (2X), and 1 µl forward/reverse primer mix (6 µM each) (KAPA Biosystems, Boston, MA, USA) were added into 48-well PCR plates for each reaction (10 μl). Thermal cycling consisted of 15 min at 95°C, followed by 40 cycles at 95°C for 15 s and 60°C for 60 s, using the StepOne System (Applied Biosystems, Foster City, CA, USA). The predicted cycle threshold (Ct) values were exported into Excel worksheets for analysis. Comparative Ct methods, normalized to GAPDH, were used to determine the gene expression levels. Sequences of oligonucleotides used as primers were as follows: Collagen1a2 (Col1a2), forward, 5′-TAGGCCATTGTGTATGCAGC-3′, reverse, 5′-ACATGTTCAGCTTTGTGGACC-3′. α-SMA, forward, 5′-GTTCAGTGGTGCCTCTGTCA-3′, reverse, 5′-ACTGGGACGACATGGAAAAG-3′. TGF-β1, forward, 5′-CGAAGCGGACTACTATGC-3′, reverse, 5′-GTTGCTCCACACTTGATTT-3′. MMP-2, forward, 5′-CTCAGATCCGTGGTGAGAT-3′, reverse, 5′-AGGCTGGTCAGTGGCTTGG-3′. MCP-1, forward, 5′-ACCGAGAGGCTGAGACTAAC-3′, reverse, 5′-CAGGTGACTGGGGCATTGAT-3′. IL-6, forward, 5′-TGGCTGAAAAAGATGGATGCT-3′, reverse, 5′-TCTGCACAGCTCTGGCTTGT-3′. TNF-α, forward, 5′-TGTAGCCCATGTTGTAGCAAACC-3′, reverse, 5′-GAGGACCTGGGAGTAGATGAGGTA-3′. IL-1, forward, 5′-CTGAGCACCTTCTTTCCCTTCA-3′, reverse, 5′-TGGACCAGACATCACCAAGCT-3′. IL-8, forward, 5′-CAGCCTTCCTGATTTCTGCAG-3′, reverse, 5′-AGACAGAGCTCTCTTCCATCAG-3′. IL-1b, forward, 5′-GCTGATGGCCCTAAACAGATG-3′, reverse, 5′-TTCTCCTGGAAGGTCTGTGG-3′. F4/80, forward, 5′-CAAGACTGACAACCAGACG-3′, reverse, 5′-ACAGAAGCAGAGATTATGACC-3′. GAPDH, forward, 5′-TCACCACCATGGAGAAGGC-3′, reverse, 5′-GCTAAGCAGTTGGTGGTGCA-3′.

### Statistical Analysis

Data are shown as the mean ± SD. Data from cell studies were evaluated by nonparametric tests. For this purpose, the Mann-Whitney U test was used to compare two independent groups. Data from animal studies were evaluated by parametric tests. For this purpose, a two-way analysis of variance was used to make multiple comparisons. Statistical analyses were performed using the SPSS program (SPSS Inc., Chicago, IL, USA), and p < 0.05 was considered to indicate statistical significance.

## Results

### The Compound BA-5 Has the Best Ability to Ameliorate TGF-β1-Induced HSC Activation Without Overt Cytotoxic Effects

**Figure 1:**
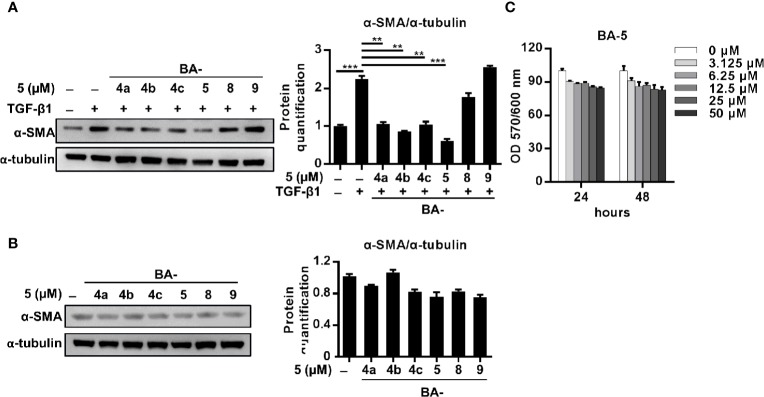
Effects of BA-5 on TGF-β1-induced LX2 cells activation. **(A)** LX2 cells were treated with 10 ng/ml TGF-β1 and 5 μM of six different barbituric acid derivatives for 24 h. The protein expression of α-SMA was assessed by the Western blot. **(B)** LX2 cells were treated with 5 μM of six different barbituric acid derivatives for 24 h. The protein expression of α-SMA was assessed by the Western blot. **(C)** LX2 cells were exposed to 0, 3.125, 6.25, 12.5, 25, and 50 μM of BA-5 for the indicated time periods, and the cell viability was assessed by using an alamarBlue assay kit. The data are expressed as the mean ± SD. Each experiment was performed in three independent replicates with similar results. **, p < 0.01; ***, p < 0.001.

Regarding the antifibrotic effects of the six different barbituric acid derivatives on TGF-β1-induced HSC activation, the protein expression level of α-SMA was analyzed. The results showed that TGF-β1-induced upregulation of α-SMA was markedly reduced after treatment with BA-5 (**Figure 1A**). While in the absence of TGF-β1 treatment, cells treated with the six different barbituric acid derivatives did not alter the protein expression of α-SMA (**Figure 1B**). To evaluate the cytotoxic effect of BA-5 on HSCs, an alamarBlue assay was performed. The results showed that after treatment with the maximum concentration of BA-5 (50 μM) for 48 h, more than 80% of the LX2 cells had survived (**Figure 1C**). Therefore, BA-5 was selected for further experiments.

### Treatment With BA-5 Inhibits TGF-β1-Induced Fibrogenesis in HSCs Cell Lines

**Figure 2:**
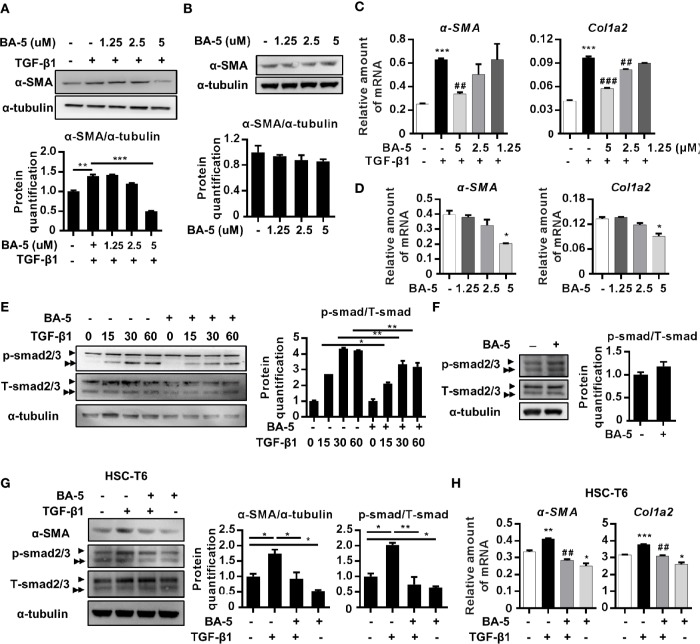
BA-5 inhibits TGF-β1 induced HSCs activation in both LX2 and HSC-T6 cells. **(A–D)** LX2 cells were treated with or without 10 ng/ml TGF-β1 and 0, 1.25, 2.5, and 5 μM of BA-5 for 24 h. Western Blot and real-time PCR were used to evaluate the protein and mRNA expression of α-SMA and Col1a2. **(E)** LX2 cells were treated with 5 μM of BA-5 for 24 h and then subjected to 10 ng/ml TGF-β1 for the indicated time periods, and the lysates were analyzed by Western blot and quantification to detect smad2/3 phosphorylation (►, smad2, ►►, smad3). **(F)** LX2 cells were treated with 5 μM of BA-5 for 24 h and the lysates were analyzed by Western blot and quantification to detect smad2/3 phosphorylation. **(G)** HSC-T6 cells were treated with or without 5 μM of BA-5 and 10 ng/ml TGF-β1 for 24 h. Western Blot was used to evaluate the protein expression of α-SMA. For detection of smad2/3 phosphorylation, HSC-T6 cells were treated with 5 μM of BA-5 for 24 h and then subjected to 10 ng/ml TGF-β1 for 30 min, and the lysates were analyzed by Western blot. **(H)** HSC-T6 cells were treated with or without 5 μM of BA-5 and 10 ng/ml TGF-β1 for 24 h. Real-time PCR were used to evaluate the mRNA expression of α-SMA and Col1a2. *, p < 0.05; **, p < 0.01; ***, p < 0.001 vs. white bar. ^##^p < 0.01; ^###^p < 0.001 vs. black bar. Data are shown as mean ± SD. Each experiment was performed in three independent replicates with similar results.

To delineate the effect of BA-5 on the inhibition of HSC activation, the expression levels of α-SMA and Col1a2 were analyzed. BA-5 significantly inhibited TGF-β1-induced α-SMA and Col1a2 protein and mRNA expression in HSCs in a dose-dependent manner (**Figures 2A, C**). While BA-5 alone administration showed no effect on changing the protein expression levels of α-SMA (**Figure 2B**). However, the mRNA expression of α-SMA and Col1a2 were reduced after treated with 5 μM of BA-5 (**Figure 2D**). The TGF-β1-induced phosphorylation of smad2/3 was diminished in BA-5-treated LX2 cells compared with that of the control (**Figure 2E**). While in the absence of TGF-β1 treatment, cells treated with BA-5 showed no change on the protein expression of phosphorylated-smad2/3 (**Figure 2F**). To confirm the effect of BA-5 on HSCs cells, we further used an immortalized strain of rat HSCs cell line, HSC-T6, in the experiment. The result showed that BA-5 significantly inhibited TGF-β1-induced α-SMA and phosphorylated-smad2/3 protein expression in HSC-T6 cells, while BA-5 alone treatment also reduced the protein expression compared with that of the control (**Figure 2G**). Besides, BA-5 treatment significantly inhibited TGF-β1-induced α-SMA and Col1a2 mRNA expression in HSC-T6 cells while BA-5 alone administration also reduced the mRNA expression of α-SMA and Col1a2 (**Figure 2H**). These results demonstrate that BA-5 exhibits an inhibitory effect on TGF-β1-induced HSC activation in both LX2 and HSC-T6 cells.

### BA-5 Reverses LPS-Induced BAMBI Downregulation and Inhibits NF-κB Pathway Activation and p65 Nuclear Translocation in HSCs

**Figure 3:**
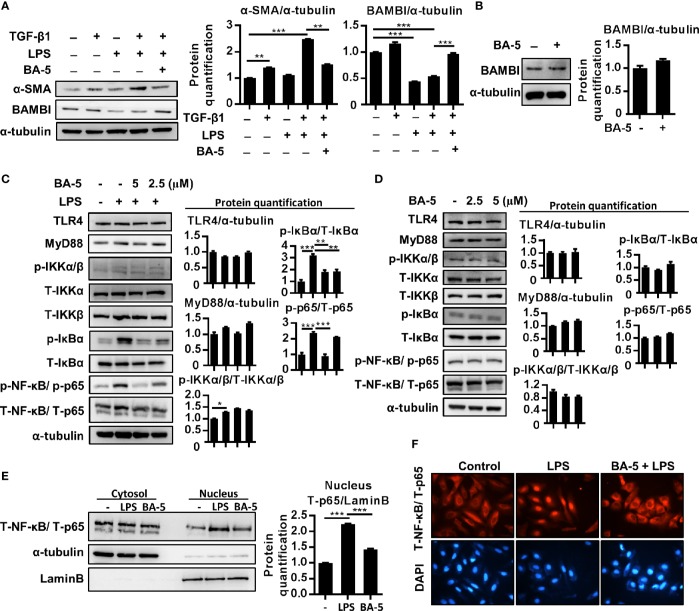
BA-5 reversed LPS-induced BAMBI downregulation and inhibits NF-κB pathway activation and nuclear translocation of p65 in HSCs. **(A)** LX2 cells were co-treated with 10 ng/ml TGF-β1, 100 ng/ml LPS, and 5 μM of BA-5 for 72 h. The protein expression of α-SMA and BAMBI were analyzed by the Western blot. **(B)** LX2 cells were treated with 5μM of BA-5 for 24 h. The protein expression of BAMBI were analyzed by the Western blot. **(C)** LX2 cells were pre-treated with 0, 2.5, and 5 μM of BA-5 for 16 h and 100 ng/ml LPS was further treated for 30 min or 48 h. Western blot was used to access the protein expression of TLR4, MyD88, and the phosphorylation of IKKα/β, IκBα, and NF-κB. **(D)** LX2 cells were treated with 0, 2.5, and 5 μM of BA-5 for 16 h and western blot was used to access the protein expression of TLR4, MyD88, and the phosphorylation of IKKα/β, IκBα, and NF-κB. **(E)** LX2 cells were pre-treated with 5 μM of BA-5 for 3 h and 100 ng/ml LPS was further treated for 1 h. Nuclear/cytosol fractionation kit was used to separate the cytosol and nuclear protein and the expression of NF-κB was analyzed by using the Western blot. **(F)** LX2 cells were pre-treated with 5 μM of BA-5 for 3 h and 100 ng/ml LPS was further treated for 1 h. Immunofluorescence assay was performed to evaluate the total p65 translocation. Each experiment was performed in three independent replicates with similar results. *p < 0.05; **p < 0.01; ***p < 0.001.

Since LPS is another important factor that influences HSC activation by downregulating the TGF-β1 pseudoreceptor-BAMBI (Seki et al., 2007), the effect of BA-5 on BAMBI expression was also analyzed. The results showed that co-treatment with TGF-β1 and LPS significantly elevated the expression level of α-SMA in HSCs, whereas treatment with BA-5 reversed LPS-induced BAMBI downregulation and significantly inhibited α-SMA expression (**Figure 3A**). In contrast, BA-5 alone treatment did not affect the protein expression of BAMBI compared with that of the control (**Figure 3B**). To investigate the anti-inflammatory effect of BA-5 on HSCs, LPS-induced TLR4-MyD88-NF-κB signaling pathway activation was analyzed. The protein expression of TLR4 and MyD88 showed no change after treatment with LPS and BA-5. On the other hand, BA-5 administration ameliorated LPS-induced IκBα and NF-κB phosphorylation, while phosphorylated-IKKα/β remained unchanged (**Figure 3C**). In the absence of TGF-β1 treatment, cells treated with BA-5 showed no effect on the regulation of TLR4-MyD88-NF-κB signaling pathway activation (**Figure 3D**). Moreover, treatment with BA-5 significantly inhibited LPS-induced total-p65 nuclear translocation, and these results were confirmed by immunofluorescence staining (**Figures 3E, F**). These results show that BA-5 inhibits LPS-induced NF-κB pathway activation.

### BA-5 Inhibits LPS-Induced Chemokine, Inflammatory Cytokine, and Adhesion Molecule Expression in HSCs

**Figure 4:**
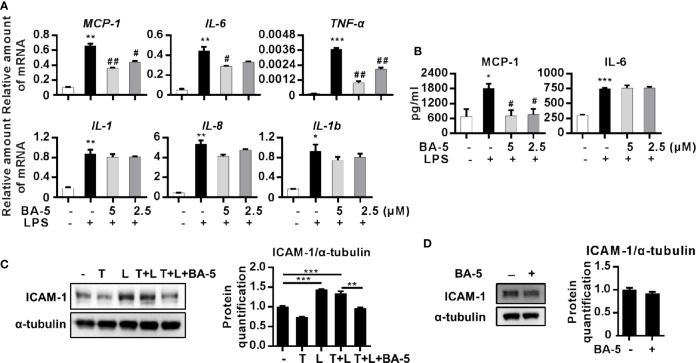
BA-5 decreases MCP-1, IL-6, and ICAM-1 expression in HSCs. **(A)** LX2 cells were pre-treated with 0, 2.5, and 5μM of BA-5 for 16 h and the cells were further treated with 100 ng/ml LPS for 3 h. Real-time PCR was used to evaluate the mRNA expression of MCP-1, IL-6, TNF-α, IL-1, IL-8, and IL-1b. **(B)** LX2 cells were pre-treated with 0, 2.5, and 5μM of BA-5 for 16 h and the cells were further treated with 100 ng/ml LPS for 9 h. DuoSet ELISA DEVELOPMENT SYSTEM (R&D Systems) was used to evaluate the protein concentration of MCP-1 and IL-6 in the cultured medium. **(C)** LX2 cells were co-treated with 10 ng/ml TGF-β1 (T), 100 ng/ml LPS (L), and 5 μM of BA-5 for 24 h. The protein expression of ICAM-1 was analyzed by the Western blot. **(D)** LX2 cells were treated with 5 μM of BA-5 for 24 h. The protein expression of ICAM-1 was analyzed by the Western blot. *p < 0.05; **p < 0.01; ***p < 0.001 vs. white bar. ^#^p < 0.05; ^##^p < 0.01 vs. black bar. Data are shown as mean ± SD. Each experiment was performed in three independent replicates with similar results.

To assess whether BA-5 could inhibit the production of LPS-induced chemokines and inflammatory cytokines, HSCs were pretreated with BA-5 and stimulated with LPS. The results showed that treatment with BA-5 significantly inhibited LPS-induced mRNA expression of MCP-1, IL-6, and TNF-α in a dose-dependent manner, while the expression of IL-1, IL-8, and IL-1b was not changed (**Figure 4A**). The secretion levels of MCP-1, IL-6, and TNF-α in the culture supernatant were detected by ELISA. The results showed that treatment with BA-5 significantly inhibited the production of LPS-induced MCP-1 in HSCs, while IL-6 was not changed (**Figure 4B**), and TNF-α was not detected (data not shown). Since LPS-induced TLR4 activation also leads to ICAM-1 upregulation in HSCs (Paik et al., 2003), the protein expression of ICAM-1 was analyzed. The results showed that treatment with BA-5 significantly inhibited LPS-induced ICAM-1 upregulation in HSCs (**Figure 4C**) while BA-5 alone administration did not change the protein expression level of ICAM-1 (**Figure 4D**). These results suggest that BA-5 treatment inhibits LPS-induced MCP-1 and ICAM-1 expression in HSCs.

### The Conditioned Medium From BA-5-Treated LX2 Cells Showed Reduced Ability to Activate RAW264.7 Macrophages

**Figure 5:**
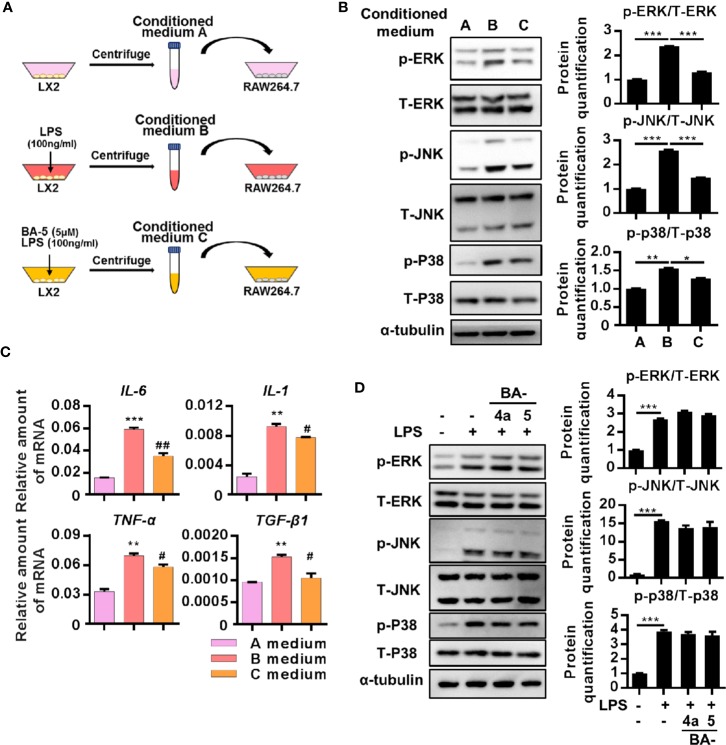
The conditioned medium from BA-5-treated LX2 possessed limited ability in activation of RAW264.7 macrophages. **(A)** Schematic diagram of the effects of BA-5 between HSCs and macrophage. **(B)** RAW264.7 cells were treated with three different conditioned mediums respectively for 24 h, and the protein phosphorylation of ERK, JNK, and p-38 were analyzed by the Western blot. **(C)** RAW264.7 cells were incubated with three different conditioned mediums respectively for 24 h. Real-time PCR was used to evaluate the mRNA expression of IL-6, IL-1, TNF-α, and TGF-β1. **(D)** RAW264.7 cells were treated with 100 ng/ml LPS and 5 μM of BA-4a and BA-5 for 24 h, and the protein phosphorylation of ERK, JNK, and p-38 were analyzed by the Western blot. *p < 0.05; **p < 0.01; ***p < 0.001 vs. white bar. ^#^p < 0.05; ^##^p < 0.01 vs. black bar. Data are shown as mean ± SD. Each experiment was performed in three independent replicates with similar results.

Since liver fibrosis is driven by the crosstalk between macrophages and activated HSCs (Wynn and Barron, 2010; Tacke and Zimmermann, 2014), we designed experiments to determine the effect of BA-5 on the interplay between macrophages and HSCs. LX2 cells were treated with or without LPS and BA-5 for 9 h, and then the conditioned medium, labeled A, B, and C, were collected and used to treat the RAW264.7 cells (**Figure 5A**). Macrophage stimulation by LPS results in the phosphorylation of the mitogen-activated protein kinase (MAPK) family (Su and Karin, 1996); thus, activation of the MAPK pathway was analyzed. The results showed that conditioned medium B from LPS-activated HSCs significantly enhanced RAW264.7 cell activation by upregulating ERK, JNK, and p38 phosphorylation (**Figure 5B**). Importantly, the phosphorylation of ERK, JNK and P38 was decreased when RAW264.7 cells were exposed to conditioned medium C (collected from BA-5-treated HSCs) (**Figure 5B**). When the MAPK pathway is activated, macrophages activate and express inflammation-related genes (Lloberas et al., 2016). BA-5-treated HSC-conditioned medium C showed reduced IL-6, IL-1, TNF-α, and TGF-β1 mRNA expression compared with that of conditioned medium B, which was collected from LPS-activated HSCs (**Figure 5C**). To determine whether BA-5 can directly inhibit macrophage activation, RAW264.7 cells were treated with LPS and compounds BA-4a and BA-5. The protein phosphorylation of ERK, JNK, and p38 showed no change in LPS- and BA-5-treated RAW264.7 cells (**Figure 5D**). These results demonstrate that BA-5-treated LX2 cells possess limited ability to activate macrophages.

### BA-5 Pretreatment Attenuates CCl_4_-Induced Liver Damage, Liver Fibrosis, and Macrophage Infiltration in Mice

**Figure 6:**
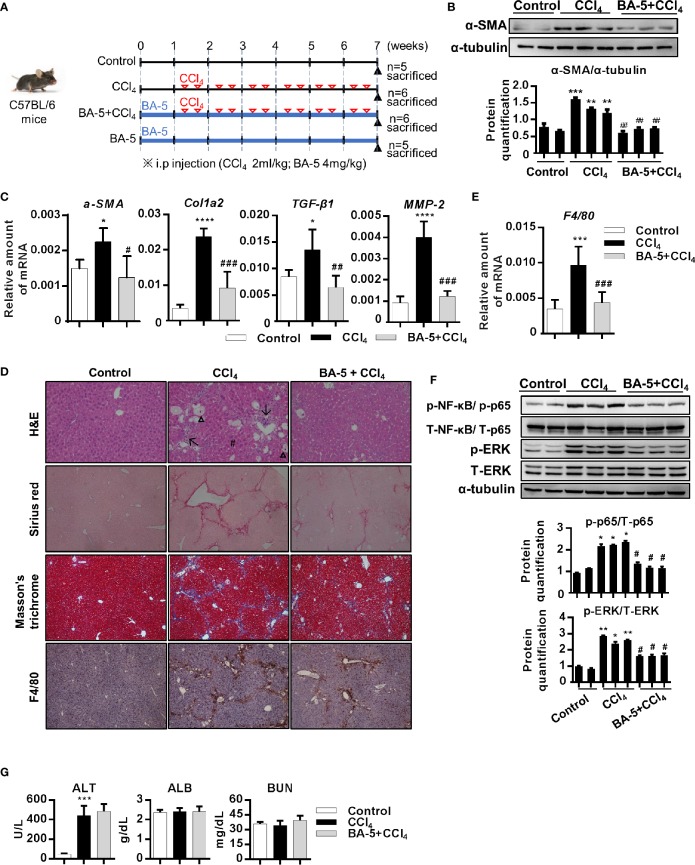
BA-5 pretreatment decreased CCl_4_-induced liver damage, liver fibrosis, and macrophage infiltration. **(A)** Study design of the *in vivo* experiment. **(B)** Representative results from Western blot analyses of α-SMA expression in three groups. **(C)** Results from QPCR analyses of α-SMA, Col1a2, TGF-β1, and MMP2 gene expression in three groups. **(D)** Representative H&E, Sirius red, Masson’s trichrome, and F4/80 IHC staining images of liver tissues from control, CCl_4_, and BA-5+CCl_4_ mice. Original magnification, ×100. Δ, fatty change; #, perinuclear vacuoles; ↑, inflammatory infiltration. **(E)** Results from QPCR analyses of F4/80 gene expression in three groups. **(F)** Representative results from Western blot analyses of T/p-p65, and T/p-ERK expression in three groups. **(G)** Serum samples were collected at the end of the experiment, and the serum ALT, ALB, and BUN values were assessed.*, p < 0.05; **, p < 0.01; ***, p < 0.001; ****, p < 0.0001 vs. white bar. ^#^p < 0.05; ^##^p < 0.01; ^###^p < 0.001 vs. black bar. Data are shown as mean ± SD.

To further correlate the *in vivo* antifibrotic effects of BA-5 with the mechanisms identified *in vitro*, a CCl_4_-induced liver fibrosis mouse model was used (**Figure 6A**). The results showed that the protein expression of α-SMA was increased after the development of CCl_4_-induced liver fibrosis, and BA-5 administration significantly reduced the protein expression of α-SMA (**Figure 6B**). The mRNA expression levels of α-SMA, Col1a2, TGF-β1, and MMP2 were significantly increased in the CCl_4_ group, while BA-5 administration resulted in significant inhibition of the expression levels of these genes (**Figure 6C**). H&E staining showed that CCl_4_ treatment induced more severe fatty change, irregular perinuclear vacuoles, and regional inflammation in mice liver (**Figure 6D**). In contrast, BA-5 treatment ameliorated CCl_4_-induced liver damage (**Figure 6D**). Collagen deposition, a marker for liver fibrosis, was assessed by Sirius red and Masson’s trichrome staining. As shown in **Figure 6D**, fibrillary collagen deposition was observed in CCl_4_-treated mice, notably, this phenomenon was attenuated in BA-5 treated mice. Macrophage infiltration in the liver was assessed by the IHC staining of F4/80, and the results showed that BA-5 administration significantly inhibited CCl_4_-induced F4/80 expression (**Figure 6D**). The mRNA expression levels of F4/80 were also analyzed, and the results showed that BA-5 administration significantly inhibited CCl_4_-induced F4/80 mRNA expression (**Figure 6E**). Furthermore, we analyzed the regulation of the NF-κB and MAPK pathways. The results showed that BA-5 administration significantly inhibited CCl_4_-induced NF-κB and ERK protein phosphorylation (**Figure 6F**). The liver function (ALT and ALB) and kidney function (BUN) values were also analyzed, and the results showed that CCl_4_ administration significantly increased ALT level while BA-5 administration did not influence the CCl_4_-induced serum values of ALT (**Figure 6G**). Besides, both CCl_4_ and BA-5 administration did not influence the serum values of ALB and BUN (**Figure 6G**). These data demonstrate that BA-5 pretreatment ameliorates CCl_4_-induced liver damage, liver fibrosis, and macrophage infiltration.

### BA-5 Administration Does Not Induce Adverse Effects in Mice

**Figure 7:**
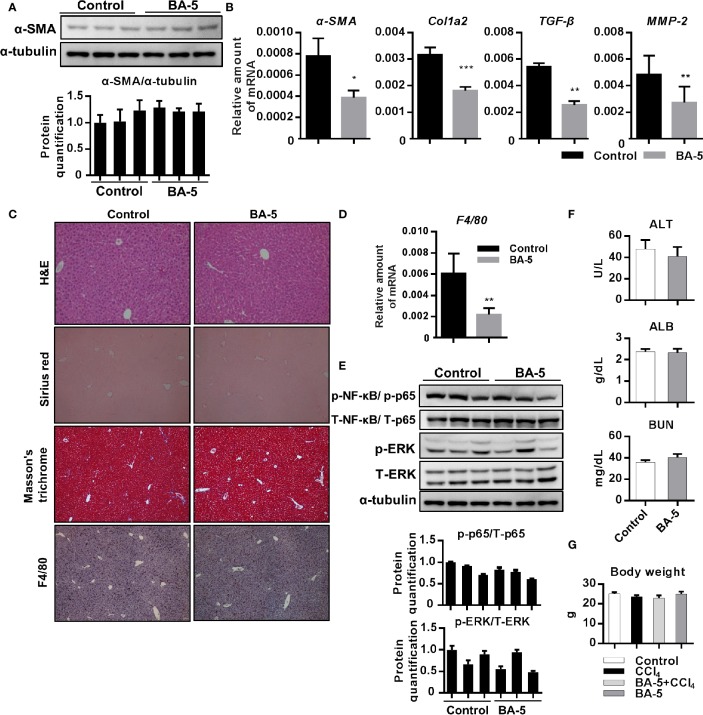
Adverse effects of BA-5 administration in mice. **(A)** Representative results from Western blot analyses of α-SMA expression and **(B)** QPCR analyses of α-SMA, Col1a2, TGF-β1, and MMP2 gene expression. **(C)** H&E, Sirius red, Masson’s trichrome, and F4/80 IHC staining of liver tissues from each treatment group. **(D)** Results from QPCR analyses of F4/80 gene expression and **(E)** Western blot analyses of T/p-p65, and T/p-ERK expression. **(F)** The serum ALT, ALB, and BUN values in each group. **(G)** The value of mouse body weight before sacrificed. *p < 0.05; **p < 0.01; ***p < 0.001 vs. black bar.

Next, we further investigate whether BA-5 alone administration will affect liver function or cause any adverse effects *in vivo*. The result showed that the protein expression of α-SMA was similar between control and BA-5 treated group (**Figure 7A**) while the mRNA expression levels of α-SMA, Col1a2, TGF-β1, and MMP2 were decreased in BA-5 administration group (**Figure 7B**). Histological liver section staining with H&E, Sirius red, and Masson’s trichrome showed no hepatic damage and fibrillary collagen deposition in BA-5-treated mice (**Figure 7C**). The mRNA expression levels of F4/80 was reduced in BA-5 administration group (**Figure 7D**) while the IHC staining of F4/80 showed that the protein expression of F4/80 remained unchanged compared with that of vehicle-treated mice (**Figure 7C**). In addition, the protein expression of phosphorylated-NF-κB and ERK were no significantly differences between BA-5-treated and vehicle-treated mice (**Figure 7E**). The serum values of ALT, ALB, and BUN were not significantly different between BA-5-treated and vehicle-treated mice (**Figure 7F**). To assess the *in vivo* toxicities mediated by BA-5, mouse body weights were measured before sacrificed, and the results showed that the mouse body weights were similar among the various groups (**Figure 7G**).

## Discussion

**Figure 8:**
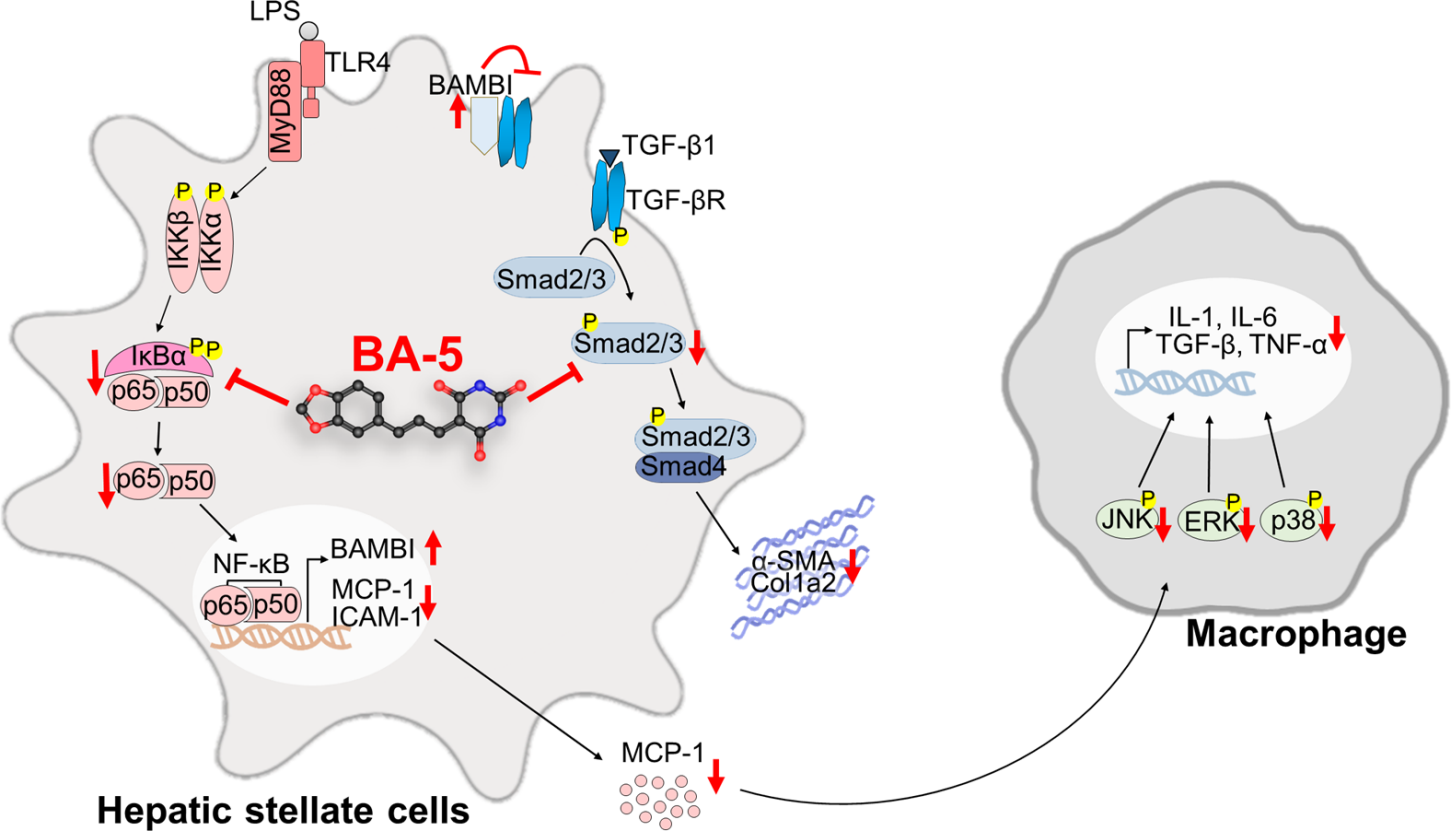
Compound BA-5 inhibits HSCs activation through blocking both TGF-β1 and LPS-induced NF-κB signaling pathways and further inhibits macrophage recruitment and activation. BA-5 inhibited TGF-β1-induced α-SMA, col1a2, and phosphorylated-smad2/3 expression in HSCs. Furthermore, BA-5 treatment enhanced BAMBI expression and inhibited LPS-induced IκBα and NF-κB phosphorylation, NF-κB nucleus translocation, MCP-1 secretion, and ICAM-1 expression. And then, reduced the activation of macrophage (RAW264.7) by inhibiting the protein phosphorylation of ERK, JNK, and p38.

Liver fibrosis is the first step toward the progression of cirrhosis and hepatocellular carcinoma (Friedman, 2008). Despite our increasing understanding of the disease, there are no effective and clinically approved antifibrotic therapies available (Trautwein et al., 2015). Thus, the identification of a new drug for treating liver fibrosis is urgently needed (Levada et al., 2019). In this study, we generated six different barbituric acid derivatives and selected compound BA-5 as the candidate with the most potential. From the *in vitro* experiments, we found that BA-5 treatment significantly inhibited TGF-β1-induced α-SMA, Col1a2, and phosphorylated-smad2/3 expression and effectively increased expression of the TGF-β1 pseudoreceptor BAMBI. Moreover, treatment with BA-5 decreased LPS-induced IκBα and NF-κB phosphorylation, NF-κB nuclear translocation, MCP-1 protein secretion, and ICAM-1 protein expression in HSCs. In addition, the conditioned medium from BA-5-treated HSCs showed reduced ability to promote macrophages activation. In the *in vivo* experiment, BA-5 administration reduced CCl_4_-induced liver damage, liver fibrosis, and F4/80 expression without any adverse effects in mice (**Figure 8**).

Barbituric acid was first prepared by Adolf Von Baeyer in 1864 from the fusion of urea and malonic acid (Baeyer, 1864). The derivatives of barbituric acid, known as barbiturates, have a special place in pharmaceutical chemistry because of the susceptibility to rapid metabolic attack and subsequent degradation of the compounds by acidic hydrogen within the body (Adams, 1958). Substances of the barbituric acid group have been used therapeutically for many years. Barbiturates are known as central nervous system depressants and are mainly used in the treatment of anxiety and epilepsy and to induce sleep and anesthesia (Shonle and Moment, 1923; Sandberg, 1951; Andrews et al., 1979; Srivastava and Kumar, 2004). Recently, it was found that barbituric acid derivatives possess anticancer effects (Singh et al., 2009; Dhorajiya et al., 2014). In liver diseases, barbituric acid derivatives alleviate the development of non-alcoholic fatty liver disease by regulating adipocytokine expression and preventing insulin resistance (Ma et al., 2011; Zheng et al., 2011). However, the effect of barbituric acid derivatives on liver fibrosis has not been reported until now. In this study, we synthesized a series of barbituric acid derivatives and investigated the detailed antifibrotic mechanisms. Our results demonstrated that the compound BA-5 not only exerts an anti-inflammatory effect on the HSCs and attenuates subsequent macrophages activation, but also inhibited CCl_4_-induced liver fibrogenesis. Notably, BA-5 alone treatment demonstrated the ability in inhibiting the mRNA expression of fibrogenesis makers *in vitro* and *in vivo* (**Figures 2C, D, H**, and **Figure 7B**). In contrast, the protein levels of α-SMA and phosphorylated-smad2/3 were not significantly changed except in the HSC-T6 cell lines (**Figure 2G**). This study provides new insight into the potential of barbituric acid derivatives in treating liver fibrosis. And further study is required to clarify the inhibitory effects of BA-5 at transcription level.

Liver fibrosis is tightly associated with chronic liver inflammation. After liver injury, intestinal mucosal permeability and bacterial translocation are increased and lead to elevated LPS levels in the portal and systemic circulation (Ponziani et al., 2018). A previous study found that LPS was increased in hepatic fibrosis mouse models and cirrhosis patients (Wiest and Garcia-Tsao, 2005; Pradere et al., 2010). The molecular link between liver fibrosis and inflammation was shown to be TLR4 signaling, which promotes HSC activation and modulates TGF-β1 signaling (Seki et al., 2007). Therefore, the investigation of potential inhibitors of both TLR4 signaling and TGF-β1 signaling represents an attractive strategy for the treatment of liver fibrosis. Our results demonstrated that the compound BA-5 exerts a suppressive effect on the TGF-β1 signaling pathway by inhibiting α-SMA, Col1a2, and phosphorylated-smad2/3 expression (**Figure 2**). In addition, BA-5 not only directly suppressed TGF-β1-induced HSC activation but also inhibited LPS-enhanced sensitivity of HSCs to TGF-β1. Similar to Seki el al. (Seki et al., 2007), we found that LPS-induced TLR4 pathway activation could sensitize HSCs to TGF-β1 stimulation through downregulation of the TGF-β1 pseudoreceptor BAMBI (**Figure 3A**). BA-5 was able to inhibit the LPS-mediated sensitization of LX2 cells to TGF-β1. The mechanism by which BA-5 inhibits LPS-mediated profibrogenic effects might relate to the findings that BA-5 suppressed the activation of TLR4 downstream signal transduction, including phosphorylation of IκBα and NF-κB nuclear translocation (**Figure 3**). These results indicate that the anti-inflammatory and antifibrogenic effects of BA-5 may be associated with the inhibition of both the TGF-β1 and NF-κB signaling pathways. NF-*κ*B contributes to the survival and development of the cells that carry out immune responses in mammals (Zhang et al., 2017). Although there are some FDA-approved drugs used in treating multiple myeloma with the strategy to suppress NF-κB, theses agents have been thwarted by side effects including nephrotoxicity, neuropathy, and the malignancy (Mina et al., 2016). In previous study, a barbituric acid derivative was found to posse the ability to block IL-2 production in activated Jurkat T cells (Xu et al., 2016). In our animal study, we did not find any body weight lost, liver and kidney damage in BA-5 administrated mice group (**Figure 7**). However, we cannot exclude the possibility of disrupting the innate immune response or affecting immunomodulatory activity while using BA-5. Therefore, further studies are required in the future to confirm the safety of using this agent.

A previous study found that fibrogenesis is a dynamic process that is tightly regulated by macrophage-stellate cell interactions (Chang et al., 2013). Crosstalk between macrophages and stellate cells drives fibrogenic processes *via* juxtacrine or paracrine signaling pathways (Holt et al., 2010). In the liver, HSCs regulate leukocyte trafficking and activation through secretion of MCP-1 and overexpression of ICAM-1 (Tacke and Zimmermann, 2014). It has been found that the expression of chemokine (MCP-1 and IL-8) and adhesion molecule (ICAM-1 and VCAM-1) genes are regulated by NF-κB (Baeuerle and Henkel, 1994; Tacke and Zimmermann, 2014). Moreover, patients with hepatitis (Leifeld et al., 2003) and experimental animal liver injury models (Paik et al., 2003) have elevated expression levels of MCP-1. Our data showed that BA-5 treatment inhibited LPS-induced mRNA expression and secretion of MCP-1 and protein expression of ICAM-1 (**Figure 4**). To further explore the interplay between HSCs and macrophages, we used a conditioned medium study, and the results demonstrated that LPS-activated HSCs drove the activation of macrophages, and BA-5 treatment reversed the effects (**Figure 5**). Furthermore, in CCl_4_-induced liver fibrogenesis mouse models, we showed that BA-5 not only inhibited liver fibrosis but also reduced macrophage infiltration *in vivo* (**Figure 6**).

In conclusion, our research showed that the barbituric acid derivative BA-5 efficiently reduces hepatic stellate cells activation through inhibition of the NF-κB and TGF-β1 signalling pathways and further alleviates liver fibrosis formation in CCl_4_-induced mouse models. Altogether, these results suggest the dual therapeutic potential of BA-5 as an inhibitor of both liver fibrosis and inflammation pathways.

## Data Availability Statement

All datasets generated for this study are included in the article/supplementary material.

## Ethics Statement

The animal study was reviewed and approved by Institutional Animal Care and Use Committee of Taipei Medical University (LAC-2017-0476).

## Author Contributions

Conceptualization, Y-JL and Y-CT. Methodology, C-LL, M-HH, and T-LC. Validation, M-HH. Formal analysis, F-MS and Y-HW. Data curation, Y-CT and M-HH. Writing—original draft preparation, Y-HW and M-HH. Writing—review and editing, F-MS and C-LL. Supervision, Y-JL.

## Funding

This study was partially supported by a grant from the Ministry of Science and Technology of the Republic of China (MOST 107-2320-B-038-021 and 108-2628-B-038-004).

## Conflict of Interest

The authors declare that the research was conducted in the absence of any commercial or financial relationships that could be construed as a potential conflict of interest.
